# The Influence of Injectable Platelet-Rich Fibrin on the Clinical Parameters and the Levels of MMP-8 in the GCF in Non-Surgical Treatment of Periodontitis—Randomized Trial

**DOI:** 10.3390/jfb16060202

**Published:** 2025-06-01

**Authors:** Anna Skurska, Marek Chwiedosik, Anna Justyna Milewska, Robert Milewski, Michał Pawłowski, Jennifer Alberichi, Violetta Dymicka-Piekarska, Martina Stefanini

**Affiliations:** 1 Department of Integrated Dentistry, Medical University of Bialystok, ul. M. Skłodowskiej-Curie 24A, 15-276 Bialystok, Poland; anna.skurska@umb.edu.pl; 2 Specialist Dental Clinic, Medical University of Bialystok, ul J. Kilińskiego 1, 15-089 Białystok, Poland; 3 Department of Biostatistics and Medical Informatics, Medical University of Bialystok, ul. Szpitalna 37, 15-295 Bialystok, Poland; anna.milewska@umb.edu.pl (A.J.M.); robert.milewski@umb.edu.pl (R.M.); michal.pawlowski@umb.edu.pl (M.P.); 4 Department of Comprehensive Rehabilitation of Medium and High Complexity, School of Dentistry, University of Buenos Aires, Marcelo Torcuato de Alvear 2142, C1122 Cdad, Autónoma de Buenos Aires C1122AAH, Argentina; jennifer.alberichi@odontologia.uba.ar; 5 Department of Clinical Laboratory Diagnostics, Medical University of Bialystok, ul. Waszyngtona 15, 15-269 Bialystok, Poland; violetta.dymicka-piekarska@umb.edu.pl; 6 Department of Biomedical and Neuromotor Sciences, Bologna University, Piazza di Porta S. Donato St. 2, 40-127 Bologna, Italy; martina.stefanini2@unibo.it

**Keywords:** periodontitis, i-PRF, MMP-8, SRP

## Abstract

Background: This study evaluates non-surgical therapy combined with injectable platelet-rich fibrin (i-PRF) on the clinical parameters and the levels of matrix metalloproteinase-8 (MMP-8) in the gingival crevicular fluid (GCF) in patients with periodontitis. Methods: Forty subjects diagnosed with periodontitis were randomly divided into two groups. In the test group, scaling and root planing (SRP) was performed with the subsequent application of i-PRF into periodontal pockets, while in the control group SRP was performed alone. Clinical examination was performed before and 1, 3 and 6 months after treatment. For MMP-8 level determination, the ELISA method was used. Results: In both groups, a statistically significant reduction in full mouth probing depth (FMPD), full mouth clinical attachment level (FMCAL), full mouth bleeding on probing (FMBOP), full mouth plaque index (FMPI) and full mouth marginal bleeding index FMMBI (*p* < 0.001) was observed. In the test group, the reduction in FMPD and FMBOP was statistically significantly greater than in the control group (*p* = 0.049 and *p* < 0.001, respectively). A significantly greater reduction of probing depth (PD) and clinical attachment level (CAL) in pockets > 5 mm between baseline and examination after 3 and 6 months was noted in the test group. The level of MMP-8 was statistically significantly reduced in both groups (*p* = 0.007 and *p* = 0.009). Conclusions: SRP significantly improves the clinical parameters and reduces MMP-8 levels in patients with periodontitis. Addition of i-PRF may further enhance the positive effects of periodontal treatment on clinical parameters, without significant influence on MMP-8 levels. The results of the research require confirmation in a more homogeneous group, taking into account the elimination of the specified limitations.

## 1. Introduction

Periodontitis is a progressive and destructive inflammatory disease that affects the teeth-supporting tissues. It is characterized by loss of attachment, the formation of periodontal pockets, gingival bleeding and formation of bone defects observed in radiological examination [1,2]. Periodontitis is a complex disease taking into account the interaction between subgingival microflora, the body’s immune response and environmental factors [3,4]. Bacterial biofilm is considered the main etiological factor causing gingivitis and leading to the destruction of periodontal tissues [3,4,5].

In periodontitis, an increased number of periopathogens in the subgingival area triggers defense mechanisms associated with the production of inflammatory factors and in-creased amounts of gingival crevicular fluid (GCF) [5]. As a result of inflammation and increased expression of proinflammatory cytokines, such as interleukin 1 (IL-1), tumor necrosis factor α (TNF-α) and interleukin 6 (IL-6), periodontal tissues are destroyed [6]. In the GCF of patients with periodontitis, compared to healthy individuals, increased levels of IL-1, IL-6, interleukin 8 (IL-8), TNF-α, receptor activator for nuclear factor κB ligand (RANKL) protein and matrix metalloproteinases (MMPs) are observed [7,8]. It is believed that the level of proteinases plays an important role in the regulation of tissue turnover and reflects the advancement of periodontal changes [9,10].

Matrix metalloproteinases are proteolytic metal-dependent endopeptidases capable of degrading the components of the extracellular matrix (ECM), including collagen, elastin, gelatin, matrix glycoproteins and proteoglycans [9,10,11]. Their ubiquitous presence in many cells suggests their importance in various biological processes, such as immune response, wound healing or neoplastic processes [11,12,13,14]. One of the best known and studied matrix metalloproteinases is MMP-8 (collagenase 2; neutrophil type) [15,16,17]. MMP-8 is a 75 kDa enzyme present mainly in the specific granules of neutrophil granulocytes. However, its presence has also been found in chondroblasts, as well as in gingival and periodontal fibroblasts [18,19,20]. An important biological function of MMP-8 in the periodontium is to facilitate the migration of leukocytes, especially neutrophils, from the circulation to the periodontal pocket [21]. The studies by Golub et al. [22] and Ingman et al. [23] first showed that MMP-8 is the main collagenase present in situ in the gingiva and gingival crevicular fluid of patients with chronic periodontitis and most likely initiates the process of tissue destruction at sites of active inflammation. The literature also showed that the increase in MMP-8 levels is a result of increased oxidative stress caused by neutrophil-derived myeloperoxidase and metabolites of *Porphyromonas gingivalis* and *Treponema denticola* [24]. The expression and activation of MMP-8 therefore reflect the severity of inflammatory changes as well as the degree of tissue degradation in periodontitis [25]. Moreover, a recently published systematic review and meta-analysis showed that the level of this biomarker (MMP-8) is clinically most useful in the diagnosis of periodontitis, regardless of whether the patient is a smoker [16,17].

Due to the infectious nature of the disease, treatment of periodontitis focuses largely on eliminating bacteria from the oral cavity. Non-surgical treatment involves mechanically breaking down the structure of the bacterial biofilm, thereby reducing the number of pathogens present in the periodontal pockets [26,27,28]. Subgingival calculus deposits, which are also eliminated during these procedures, constitute an additional place for plaque accumulation and mechanically irritate the periodontal tissues [28]. The basis of therapy and the “gold standard” of treatment is scaling and root planing (SRP) [26,27]. By eliminating periopathogens and reducing inflammation, the pockets become shallower, and swelling and bleeding are eliminated, thereby improving periodontal health [28,29,30]. Although SRP is a well-known and proven method of treatment for periodontitis, there is a constant search for means to improve the treatment outcome. Autologous blood-derived platelet concentrates are easily available and relatively inexpensive to obtain. Platelet concentrates are produced by centrifugation of a sample of the patient’s peripheral blood [30,31]. Platelet-rich fibrin (PRF) is a second-generation platelet specimen described by Choukroun et al. [32,33]. It is obtained by centrifuging blood in glass tubes or plastic tubes lined with a glass coating without the addition of anticoagulants. The preparation creates a three-dimensional fibrin network, containing platelets and leukocytes, growth factors and cytokines. The fibrin network allows slower degradation and longer release of growth factors at the site of deposition [34]. Over the years, Choukrun et al. have developed techniques for obtaining different forms of PRF, depending on the centrifugation protocol used. Injectable PRF (i-PRF) is a liquid form of the preparation, obtained from blood collected in test tubes made of specially developed polycarbonate [35].

After centrifugation, i-PRF contains liquid fibrinogen and thrombin that have not been converted to fibrin. i-PRF transforms into its solid, three-dimensional structure after 10–15 min of centrifugation [36,37]. The obtained consistency allows for the specimen to be administered as an injection, thanks to which it is possible to apply it to periodontal pockets after the SRP procedure and obtain a stable dressing with significant therapeutic properties [38,39].

The first two weeks are crucial for epithelial healing [40]. Growth factors and numerous mediators released from the PRF fibrin network contribute to the healing process in the periodontium by regulating the main pathways of collagen synthesis and tissue repair [41]. The results obtained by Gizem Torumtay Cin et al. [42] confirmed the beneficial effect of i-PRF on the healing processes in the periodontium. The administration of i-PRF increased the level of VEGF and IL-10 and reduced the level of TNF-α in GCF, which resulted in limiting the inflammatory process and inducing the repair phase in tissues. Scientific reports confirm the influence of PRF on the stimulation of angiogenesis, proliferation, migration, adhesion and differentiation of many cells, as well as the activation of cell signaling. Moreover, the ability of PRF to reduce inflammation, suppress osteoclastogenesis and increase the expression of many growth factors in mesenchymal cells has been confirmed [37,43]. Recently published papers also documented the effect of i-PRF on the polarization of macrophages from the proinflammatory M1 phenotype to the anti-inflammatory M2 phenotype and thus confirmed the ability of i-PRF to reduce inflammation not only in the cytokine pathway [44].

Clinicians performing standard procedures consider enriching them with various additional means that can improve the therapeutic effect. The guidelines presented in the last workshop of the EFP highlight the validity of different treatment methods and importance of scientific evidence in the clinical decision-making process [45]. However, they do not cover the use of platelet concentrates, i.e., platelet-rich fibrin (PRF), in the non-surgical treatment of periodontitis, and the topic of PRF is extremely interesting. Currently, published studies present the use of various forms of RPF, including i-PRF, in various fields of medicine and dentistry [46]. Nowadays, PRF is a well-documented and recognized material used in many areas of periodontology and many adopt it as the standard of clinical practice [47,48,49,50].

The use of i-PRF in healing processes and modulation of the inflammatory paths is of interest and is the subject of many studies. It is successfully used in procedures in the field of periodontal and dental surgery or orthopedics [37,51]. However, the effectiveness of i-PRF in non-surgical periodontal treatment remains controversial [52,53]. The need for further research and standardization of procedures using various forms of PRF is still emphasized [52,53]. There is also a noticeable contradiction in the results obtained by different authors [53]. Therefore, the aim of the study was to assess the effect of i-PRF on clinical parameters, as well as the level of metalloproteinase-8 in patients with periodontitis.

## 2. Materials and Methods

### 2.1. Sample Size

**Figure 1:**
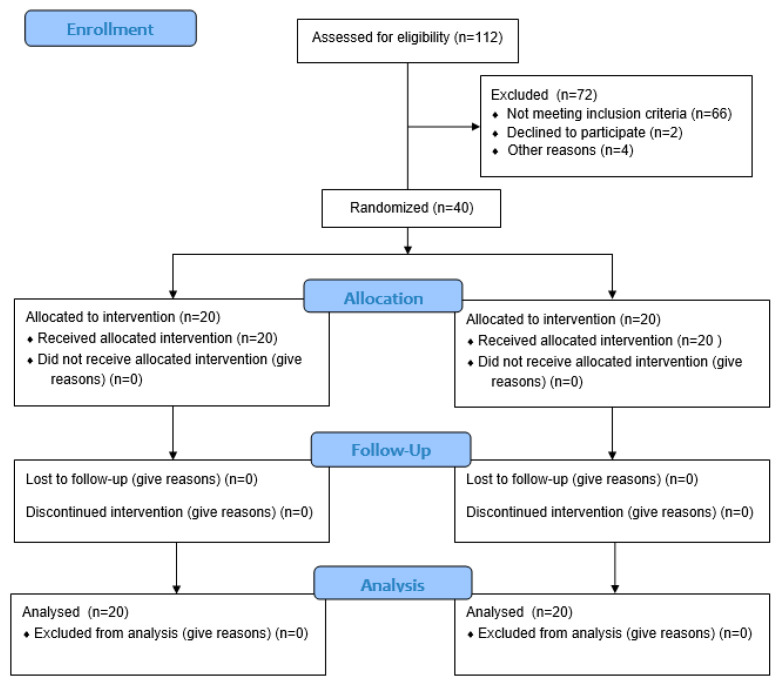
Study flowchart.

The primary study outcome was the change in the CAL. The sample size was calculated from the research results obtained by Vukovic et al. [54] to detect a clinically significant difference of 0.5 mm in the CAL between the two therapeutic procedures. In order to achieve a study power of 80%, with an α error of 0.05, assuming that the standard deviation (SD) was 0.44 mm, a required sample size of 19 patients per group was calculated. To account for a 5% dropout that could occur during the course of the study, 40 patients seeking periodontal therapy were considered for recruitment. Screening continued until a total of 40 patients (20 per group) were enrolled (Figure 1).

### 2.2. Study Population and Experimental Design

Forty patients (26 women and 14 men) aged 31–75 years treated at the Department of Periodontal and Oral Mucosa Diseases, Medical University of Białystok, Poland between April 2022 and June 2023 were qualified for the study. Based on the clinical and radiological examination, periodontitis was diagnosed at stage II or III grades A and B [55]. The protocol of the study was registered in Clinicaltrials.gov (NCT06920849) and conducted in accordance with the Helsinki Declaration of 1975, as revised in 2013, and was reviewed and approved by the local ethical committee (Bioethics Committee of the Medical University of Białystok, APK.002.122.2022, date 17 February 2022). The study was designed as a single-center, randomized, prospective trial and was conducted in accordance with the CONSORT statement. Informed consent was obtained from all subjects involved in the study.

The inclusion criteria for patients were: clinical and radiological features of periodontitis in stage II or III; presence of at least 4 teeth and at least 2 pockets (localized not on the same tooth) with a depth of at least 5 mm in each quadrant.

The exclusion criteria for patients were as follows: periodontal treatment within 3 months prior to the study; antibiotic therapy within 3 months prior to the study; smoking; presence of systemic diseases affecting periodontal healing, i.e., immunosuppressive diseases, diabetes, osteoporosis, AIDS, hypertension treated with calcium channel blockers; use of steroids or other immunosuppressive drugs; coagulation disorders and use of drugs affecting its mechanisms; pregnant and breastfeeding women.

### 2.3. Clinical Examinations

All included patients underwent the first step of treatment including: oral hygiene instructions, professional mechanical plaque removal (PMPR), control of plaque retentive factors (i.e., removing filling overhangs) and discussing the risk factors of periodontal disease. Oral hygiene was reinforced at each following visit. All appointments were carried out by one operator (A.S.)

A clinical examination was performed using a periodontal probe (PCP UNC15, Hu-Friedy, Chicago, IL, USA). Measurements were rounded to 0.5 mm. It was performed four times: before treatment and 1, 3 and 6 months after treatment by the same experienced and calibrated examiner. The examiner was blinded with respect to the treatment procedure performed (M.C.). Basic clinical parameters were determined:

- Full mouth plaque index (FMPI, [56]) and full mouth marginal bleeding index (FMMBI) at four aspects of each tooth.
- Full mouth bleeding on probing (FMBOP [57]), full mouth pocket depth (FMPD), full mouth clinical attachment level (FMCAL), full mouth gingival recession (FMGR) at six points of each tooth: mesio-vestibular (mv), mid-vestibular (v), disto-vestibular (dv), mesio-lingual (ml), mid-lingual (l), disto-lingual (dl).
- In periodontal pockets equal or deeper than 5 mm clinical parameters were determined as follows: pocket depth (PD) from the gingival margin to the bottom of the sulcus, gingival recession (GR) from the CEJ to the most apical extension of gingival margin, clinical attachment level (CAL) from the CEJ to the bottom of the sulcus.

### 2.4. Clinical Procedure

The test group consisted of 20 participants (11 women and 9 men; aged 31–75 years) who underwent the scaling and root planing (SRP) procedure with i-PRF administration into the pockets. In the control group also consisting of 20 people (15 women and 5 men; aged 35–62 years) the SRP procedure was performed alone. The therapeutic procedure was performed under local anesthesia (Septanest 200, Septodont, Paris, France), using an ultrasonic device (EMS Piezon, Tip PS, EMS, Nyon, Switzerland) and hand instruments (Gracey currettes (SMS), Hu-Friedy, Chicago, IL, USA) by one operator (A.S.)

In the test group, i-PRF was prepared using a device available at the Department of Periodontal and Oral Mucosa Diseases (PRF Duo Quattro Centrifuge, Choukroun Pro-cess For PRF, Nice, France). After the SRP procedure, allocation was performed according to a randomization table prepared by a statistician, and i-PRF was administered into the pockets by one trained operator (A.S.)

Blood collection and injectable platelet-rich fibrin (i-PRF) preparation procedure.

Venous blood was collected from test group patients after the SRP procedure. After changing the patient’s position to a sitting position and assessing the ulnar bend, the puncture site was selected. It was the cephalic vein, the basilic vein or the median ulnar vein. Then, tourniquets were placed 7–10 cm above the puncture site, taking care not to exceed one minute for the clamping. The ulnar bend was disinfected using an antiseptic (Skinsept Pur, Ecolab sp z o.o., Krakow, Poland) and the skin above the puncture site was stretched to immobilize the vein. The needle was inserted into the lumen of the vessel at an angle of 15–30°, with the bevel facing upwards until blood appeared in the tube (Safe GBO Butterfly Needle with Vacuette Holder (Grainer), 21G (0.8 × 19 mm) with a 7.5” (19 cm) tube, ref:450085). Blood was collected into 2 S-PRF sticky tubes marked in green (S-PRF Sticky, ProCell, Dermoaroma Italy Sal, Rome, Italy). The tubes were then placed in a centrifuge in opposite slots. The i-PRF program was set to 3 min of centrifugation at 700 rpm. After centrifugation, i-PRF was collected with a sterile injection needle and syringe. After changing the injection needle to a sterile blunt one, i-PRF was administrated to periodontal pockets from the bottom of the pocket to the gingival edge.

Postoperatively, patients from both groups used a rinse containing 0.2% chlorhexidine (Eludril, Pierre Fabre Laboratories, Paris, France) twice daily for 2 weeks.

Procedure for collecting and determining the volume of periodontal crevicular fluid (GCF).

At the first visit and at 2, 4 and 12 weeks, as well as 6 months after the procedure, periodontal pocket fluid (GCF) was collected from each patient from a periodontal pocket at least 5 mm deep of the tooth identified during the first clinical examination. All the GCF samples were collected between 9 a.m. and 1 p.m. to minimize diurnal variations. After isolating the selected tooth with lignin rolls, plaque was gently removed and the tooth was gently air-dried. The fluid was collected using paper strips (Periopaper, Interstate Drug Exchange, Amityville, NY, USA). The strip was placed into the periodontal pocket to a depth of 3 mm for 30 s. The volume of fluid absorbed on the paper strip was measured using a calibrated device as the sulcus fluid flow rate (SFFR) in relative Periotron units (PU) (Periotron 8000, Oraflow R Inc., New York, NY, USA). After measurement, samples were immediately placed in Eppendorf tubes containing 200 μL of phosphate-buffered saline (PBS) and frozen at −20 °C. At subsequent visits, GCF was collected from exactly the same sites as during the initial examination.

### 2.5. Matrix Metalloproteinase-8 (MMP-8) Level Determination Procedure

MMP-8 levels in GCF were determined by the immunoenzymatic enzyme-linked immunosorbent assay (ELISA) method with the use of commercial kits (Human Total MMP-8 Immunoassay, R&D Systems, Abington, UK, catalog number DMP800B).

The surface of the microplate was covered with specific monoclonal antibodies that bound to the tested antigen (MMP-8) present in the GCF fluid. The next step was to incubate and wash out unbound antibodies and add the conjugate, i.e., enzyme-labeled antibodies (biotin). As a result of the reaction, a “sandwich” complex was formed: antibody−antigen−antibody + enzyme. The next stage was the addition of an appropriate substrate, which, as a result of the enzyme’s action, turned into a colored product. The color intensity was directly proportional to the concentration of the tested antigen. The reading was taken at a wavelength of λ = 450 nm. The ALAB PLATE READER ELISA reader was used for measurement. Results were presented in ng/mL.

### 2.6. Statistical Analysis

Due to the small numbers in the analyzed groups, it was not possible to verify the normality of the distribution, therefore non-parametric tests were used. When comparing variables, the non-parametric Mann–Whitney U test was used in the case of two groups. When comparing dependent variables, the Friedman test was used in the case of multiple variables. Pearson’s chi-square test of independence was used to check the relationship between nominal features. Statistically significant results were considered at the level of *p* < 0.05. The Statistica 13.3 package from Tibco was used in the calculations (TIBCO Software Inc. Palo Alto, CA, USA).

## 3. Results

All patients attended their scheduled appointments and none of them withdrew from participation in the project. Healing in all patients proceeded without complications. Prolonged healing was observed in one patient, but it did not require any additional intervention (Figure 1).

In the baseline examination, no statistically significant differences were found between the studied groups in any of the assessed periodontal clinical parameters, as well in age (*p* = 0.20) and gender (*p* = 0.18) distribution (Table 1).

In both groups, a statistically significant reduction in probing depth was found in the following examinations (Table 2). The difference between the groups after one and three months was statistically significant (*p* = 0.049). In both examinations, the reduction of the parameter was greater in the group in which i-PRF was additionally used after SRP.

In both study groups, a gain in clinical attachment was observed over the course of subsequent examinations. The differences that occurred over time in both groups were statistically significant (*p* < 0.001). No significant differences were found between the study groups.

In both groups, the treatment resulted in a significant increase in the dimension of gingival recession (test *p* = 0.043, control *p* < 0.001). The differences between the study groups did not reach statistical significance.

In all patients, poor oral hygiene expressed by high FMPI values was observed. In the test group, this value was 71%, and in the control group it was 73%. This parameter systematically improved, which translated into an FMPI reduction in subsequent examinations. In both the test and control groups, the change was statistically significant (*p* < 0.001). Statistical analysis showed significant differences in the plaque index values between the groups in the study after 1, 3 and 6 months (*p* < 0.001) (Table 2).

Improper hygiene also affected the values of the marginal bleeding index (FMMBI) and bleeding on probing (FMBOP) at baseline. In both groups, a successive reduction in the values of both indices was observed. In the case of both marginal bleeding and bleeding on probing, the differences that occurred over the six months were statistically significant (*p* < 0.001).

Statistical analysis also showed significant differences between the groups. In the case of bleeding on probing, such differences occurred in all examinations (*p* < 0.001).

The analysis of changes between subsequent examinations in relation to the baseline study showed the greatest reduction in probing depth between the baseline and the examination after the first month. In relation to the control group, the difference in the test group was statistically significant (*p* = 0.011).

In terms of clinical attachment level, the increase between the baseline examination and those after 1 and 6 months in the test group was also significantly greater in relation to the control group (0.53 ± 0.16 *p* = 0.003 and 0.46 ± 0.33 *p* = 0.038, respectively) (Table 3).

Analyzing the data regarding periodontal pockets equal to or deeper than 5 mm, a comparable reduction in the number of such pockets was observed in both groups (Table 4).

In both groups, there was also a significant reduction in pocket depths (*p* < 0.001). There were no statistical differences between the groups, although the numerical means decreased more in the group where SRP was used in combination with i-PRF (Table 5).

A similar trend was also observed in the case of clinical attachment level. The gain in CAL over time was significant in both groups (*p* < 0.001), but no significant differences were found between the groups.

Analysis of changes in GR showed gingival stability in the test group. In the control group, a statistically significant increase in the dimension of gingival recession was observed (*p* = 0.007). However, this did not translate into differences between the groups in follow-up examinations.

Examining the changes that occurred between the following examinations, a statistically significant difference was shown between the groups in the difference in the PD be-tween the baseline examination and the examination after six months, in favor of the SRP + i-PRF group (*p* = 0.042). The differences between the initial examination and after one month were not statistically significant but approached it (*p* = 0.053).

A similar trend was observed in the analysis of the clinical attachment gain. Here statistically significant differences were found between the baseline examination and after three months (*p* = 0.027), as well as between the baseline examination and after six months (*p* = 0.014). In both cases, the gain was greater in the group in which i-PRF was used (Table 6).

Analysis of biochemical tests showed a statistically significant decrease in the level of MMP-8 in both the test and control groups (*p* = 0.007 and *p* = 0.009, respectively). Despite significant differences in the level of MMP-8 in the examination after two weeks, they did not reach statistical significance (*p* = 0.208). The decrease in MMP-8 levels in both groups was maintained until the 6-month examination (Table 7).

The amount of periodontal pocket fluid reported as SFFR was significantly reduced after both SRP and combined therapy. These changes were statistically significant (*p* = 0.015 and *p* < 0.001), although the difference was greater after i-PRF (Table 8).

## 4. Discussion

SRP is the gold standard in the treatment of periodontitis. Although currently there is not an instrumental technique that would enable complete elimination of bacterial biofilm, mechanical debridement of the tooth surface from subgingival deposits undoubtedly results in the return of periodontal tissues to homeostasis, reduction of inflammation and improvement of clinical parameters [59,60]. Data on the effectiveness of SRP were confirmed in a recent systematic review included in the guidelines for the treatment of stage I to III periodontitis [28]. Subgingival instrumentation is effective regardless of the treatment method or choice of tools [28]. The results obtained in our study are in agreement with the results of other authors. In a clinical study involving 33 people, Ioannou et al. [61] compared the effects of non-surgical periodontal treatment using hand tools and ultrasonic tools, where the study group included patients who underwent SRP with the use of ultrasonic tools and the control group included the use of hand tools. In both groups, the probing depth was reduced by 0.9 ± 0.14 mm in the control group and 0.4 ± 0.07 mm in the study group in 6-month observations. Although the PD reduction was numerically greater in the control group, the differences between the groups did not reach statistical significance. The reduction in probing depth in the cited studies is similar to the PD reduction obtained in the control group in our study (0.35 ± 0.20 mm) [61].

While the use of PRF in dentistry is considered a standard, there are limited data in the world literature regarding the use of i-PRF in non-surgical periodontal treatment. Gizem Torumtay Cin et al. [42], in a study of 17 patients with 34 deep periodontal pockets, found that the use of i-PRF as a complement to non-surgical periodontal treatment improves the clinical effect and effectiveness of SRP. Their results overlap to some extent with the results of our study. Probing depth reduction and attachment level gain obtained in our study were not as large as those in the cited study, but, similarly to theirs, they turned out to be statistically significant. The explanation for this situation is most likely the difference in the initial parameter values. Vučković et al. [54] in a split-mouth study in 24 patients with at least two pockets with a probing depth of ≥5 mm demonstrated the effectiveness of SRP + i-PRF therapy compared to SRP, also. A follow-up examination was performed 3 months after the procedure. The differences between the sides in clinical parameters were statistically significant.

Albonni et al. [38] conducted a controlled, randomized study in the split-mouth model on a group of 15 patients, where i-PRF was administered to the pockets on the test side and saline on the control side. In a 3-month observation period no significant differences were noted between the sides. The discrepancies in the results between the cited study and our research could be explained by differences in methodology. It should be noted that the research was conducted on a generally small group, which was additionally non-homogeneous. The study group included patients with shallow and very deep pockets, and the analyses were performed on the entire group, without their division. The analyses in our study included specification of pockets with depths exceeding 5 mm, which constitute a much greater therapeutic problem than shallow pockets [62,63,64]. Moreover, according to Matuliene et al. [65], 5 mm pockets compared to 3 mm deep pockets constitute a risk factor for tooth loss with a coefficient of 7.7. Albonni et al. [38] conducted research on a group of patients which included smokers. It is important to note that smoking is one of the risk factors for periodontal progression. The relationship between smoking and periodontitis is very well known [66,67]. The severity of periodontitis depends on the number of cigarettes smoked and the duration of the addiction [66,67]. The influence of smoking on periodontal treatment, both non-surgical and surgical, has been emphasized in the literature. Numerous studies have reported statistically worse clinical results expressed by inferior reduction in the probing depth and clinical attachment gains in the group of smoking patients compared to non-smoking patients [62,68,69,70]. Also, Shunmuga et al. [39] found no benefit from the use of i-PRF in a randomized control study in the split-mouth model in 26 patients with periodontitis accompanying type II diabetes. It should be noted that the study was performed on a group of patients suffering from metabolic disease, which is a risk factor for periodontitis, also. Moreover, the relationship between diabetes and periodontal disease is bidirectional. Diabetes affects the extent and progression of periodontitis. Scientific evidence confirms that poor glycemic control is also associated with greater intensity of periodontitis. On the other hand, glycemic control and compliance with the dietary regime help limit destruction in the periodontium. Moreover, more cardiovascular complications are observed in patients with coexisting diabetes and periodontitis than in patients with diabetes and healthy periodontium [66].

There are also several studies that used other forms of PRF. Narendran et al. [71] in a split-mouth study on a group of 16 patients with periodontal pockets greater than 5 mm and smaller than 7 mm in single-rooted teeth showed a beneficial effect of the use of L-PRF on the improvement of PD and CAL after SRP in 60- and 90-day observations. The differences between the sides turned out to be statistically significant. Unfortunately, a major limitation of the study was the sample size, as only 32 pockets were included. Moreover, only pockets in single-rooted teeth were included, while obtaining comparable results in multi-rooted teeth is much more difficult [63]. Parwani et al. [72] and Al-Rihaymee i wsp. [73] used PRF in solid form in the split-mouth model. In both studies the authors found statistically significantly better clinical results on the side where PRF was used compared to the control side.

A significant number of cited studies confirm the validity of the use of i-PRF in non-surgical treatment and its importance in reducing inflammatory processes in the periodontium. This is consistent with our study. However, one aspect of periodontal parameters may undermine the effectiveness of i-PRF. We have noted statistically significant differences between groups in terms of the plaque index (PI). Similar trends in the differences in the plaque index were also found by other authors [72]. However, they did not refer to this fact in the discussion. Overall, there is a strong link between oral hygiene and periodontal health. Although there is a weak relationship between the level of supragingival plaque and the progression of periodontitis, it has been shown that, without proper oral hygiene, treatment results cannot be maintained [74]. This is because supragingival plaque leads to the formation of subgingival plaque, which in turn leads to an increase in the severity of periodontitis [75]. With the above in mind, statistical differences in the plaque index should first translate into similar differences in the marginal bleeding index, which is related to the plaque index [76]. These changes would lead to changes in the bleeding on probing values. However, such a tendency was not observed in our study. FMMBI was statistically significantly reduced in both groups, but no such significance was found between the groups. We can therefore assume that the statistical differences in FMBOP are due to the anti-inflammatory effect of i-PRF.

It is also worth noting that, despite statistically significant differences between the groups in the level of clinical parameters, i.e., FMPI and FMBOP, this did not translate into a significant difference in the levels of MMP-8 between the groups. If inflammation and plaque levels were to influence probing depths, this should also translate into MMP-8 levels. By this fact we may suppose the importance of PRF in the healing of periodontal tissues.

All presented studies assessing the effectiveness of i-PRF were performed in the split-mouth model on a group of patients no larger than 26 people. Our study is the first to be conducted on a model of two parallel groups and on a larger group of patients. Of course, the split-mouth model has its advantages. Perhaps most important is the fact that each patient serves as his own control, and as a result, much of the interindividual variability is removed. If this model is used, the area in each patient undergoing the intervention should be the same. This may be a problem in dental specialties, such as endodontics, periodontology or conservative dentistry, where clinically significant differences between the sides of the dentition very often occur. Moreover, when using treatments in the pocket, it should be borne in mind that we are dealing with continuous production of fluid (GCF), which then enters the oral cavity. Therefore, we cannot be sure that a treatment administered in one place does not, to some extent, affect other places.

The statistically significant reduction in SFFR that occurred after therapy in both the test and control groups is a phenomenon that has been widely described in the literature, and our results are consistent with those of other authors [5,16].

It is worth noting that only in one of the presented studies did the authors decide to measure biochemical parameters (IL-10, VEGF, TNF-α). Their concentrations in GCF confirmed the transition from inflammation to healing [42]. Our research is also the first to assess the level of MMP-8 as an indicator of the effectiveness of periodontitis treatment after the use of i-PRF. Biochemical parameters can be helpful in identifying disease entities and the scale of their advancement. One of the most specific for periodontitis is MMP-8. In a meta-analysis of molecular biomarkers in GCF, Arias-Bujada et al. [16] highlight MMP-8 as a diagnostic biomarker of periodontal disease, with sensitivity of 76.7% and specificity of 92%. The authors considered it the most clinically useful and effective parameter in the diagnosis of periodontitis in generally healthy people, regardless of whether the patients are smokers. Reducing the level of MMP-8 indicates a reduction of inflammation in periodontal tissues and inhibition of the destruction of the extracellular matrix [18]. This review suggests the validity of the MMP-8 assay in the GCF, which is also confirmed by the assay results in our study. The literature does not provide us with much information about changes in MMP-8 levels after the use of PRF. To our knowledge, there are no scientific reports evaluating MMP-8 after non-surgical periodontal treatment combined with PRF. Eren et al. [77] assessed the levels of MMP-8, tissue inhibitor of metalloproteinase-1 (TIMP-1) and IL-1β in the gingival crevice fluid after the treatment of single recessions. PRF was used in the study group and CTG in the control group. In the 10-day examination, the level of MMP-8 was significantly lower in the group of patients treated with PRF compared to the control group (*p* < 0.05). In the cited study no statistical differences were found between the groups in 1-, 3- and 6-month observations. The presented results showed that PRF could promote early healing by increasing TIMP-1 levels and reducing MMP-8 and IL-1β levels. The trend of changes in MMP-8 levels is similar to that reported in our study. The analysis showed a statistically significant reduction in the enzyme level in both groups, and although this decrease was numerically greater in the test group compared to the control these differences did not reach statistical significance. Our research results have some clinical implications. They support the additional use of i-PRF in the non-surgical treatment of periodontitis. Availability and simplicity of preparation and implementation make the material a promising addition to therapy, with a relatively low cost and time consumption. It is worth noting that the treatment protocol brought some improvement in cases of pockets of critical depth, which in some cases may potentially reduce the need for surgical intervention.

### Limitations

An undoubted limitation of our study is the size of the study group. It would be rea-sonable to conduct a similar study on a much larger number of patients, but due to logistical constraints, this was not possible. An additional aspect is the lack of a placebo in the study. Patients aware of being in the control group may have felt less disciplined and thus less motivated to follow the recommendations regarding maintaining high levels of oral hygiene. This potentially explains the statistically significant differences in FMPI values between groups. On the other hand, successful use of a placebo would require blood collection from each patient in the control group. Due to the fact that the collected material would have to be disposed of and exposing the patient to procedures that would not provide benefits, such a procedure was abandoned. Undoubtedly, we can also consider the lack of double blinding as a limitation, which in our research can only be explained by limitations in the work organization.

Another limitation of our research is the lack of analyses according to gender and age. Interestingly, periodontitis occurs more often in men compared to women, which indicates a possible involvement of gender in the pathogenesis of the disease [78]. Moreover, the literature indicates a relationship between sexual dimorphism and the etiology of the disease, which may influence the bacterial component and the host’s immune response, both at the innate and adaptive levels [78]. This again brings us to the issue of the validity of conducting research to a much greater extent, taking into account different factors like gender, age and longer periods of observations.

## 5. Conclusions

Non-surgical periodontal treatment in the sense of mechanotherapy significantly improves the clinical parameters of patients with periodontitis at stages II to III. It also leads to a marked reduction in the levels of SFFR and GCF MMP-8. Although the addition of i-PRF may further enhance the positive effects of periodontal treatment on clinical parameters, particularly regarding critical pockets, it did not demonstrate any additional impact on the levels of SFFR and GCF MMP-8. The results of our research require confirmation on a more homogeneous group, taking into account the elimination of the specified limitations mentioned above.

## Figures and Tables

**Table 1:** Baseline groups characteristics.

	No	Gender	Age	PD	CAL
Test SRP + i-PRF	20	11F/9M	31–75	3.07	3.41
Control SRP	20	15F/5M	35–62	3.11	3.34
*p*		NS	NS	NS	NS

**Table 2:** Clinical parameters in test and control group at baseline and 1, 3 and 6 months posttreatment. * *p* < 0.05 [58].

	SRP + i-PRF	SRP	*p*
FM PD_0_	3.07 (±0.40)	3.11 (±0.41)	0.533
FM PD_1M_	2.53 (±0.42)	2.72 (±0.39)	0.049 *
FM PD_3M_	2.53 (±0.31)	2.68 (±0.36)	0.049 *
FM PD_6M_	2.57 (±0.40)	2.76 (±0.36)	0.081
*p*	<0.001 *	<0.001 *	
FM CAL_0_	3.41 (±0.58)	3.34 (±0.53)	0.839
FM CAL_1M_	2.88 (±0.60)	2.99 (±0.59)	0.364
FM CAL_3M_	2.90 (±0.59)	3.00 (±0.59)	0.357
FM CAL_6M_	2.95 (±0.7)	3.11 (±0.60)	0.133
*p*	<0.001 *	<0.001 *	
FM GR_0_	0.34 (±0.38)	0.23 (±0.36)	0.184
FM GR_1M_	0.35 (±0.38)	0.27 (±0.16)	0.31
FM GR_3M_	0.38 (±0.41)	0.32 (±0.46)	0.364
FM GR_6M_	0.38 (±0.41)	0.35 (±0.49)	0.473
*p*	0.043 *	<0.001 *	
FMPI_0_	71.00 (±10.00)	73.00 (±11.00)	0.579
FMPI_1M_	16.00 (±6.00)	31.00 (±14.00)	<0.001 *
FMPI_3M_	19.00 (±6.00)	34.00 (±12.00)	<0.001 *
FMPI_6M_	25.00 (±10.00)	40.00 (±14.00)	<0.001 *
*p*	<0.001 *	<0.001 *	
FMMBI_0_	66.00 (±11.00)	65.00 (±17.00)	0.379
FMMBI_1M_	16.00 (±10.00)	25.00 (±15.00)	0.065
FMMBI_3M_	17.00 (±9.00)	28.00 (±17.00)	0.071
FMMBI_6M_	24.00 (±14.00)	32.00 (±19.00)	0.193
*p*	<0.001 *	<0.001 *	
FMBOP_0_	63.00 (±11.00)	66.00 (±12.00)	0.44
FMBOP_1M_	12.00 (±6.00)	28.00 (±11.00)	<0.001 *
FMBOP_3M_	14.00 (±5.00)	30.00 (±10.00)	<0.001 *
FMBOP_6M_	18.00 (±7.00)	35.00 (±11.00)	<0.001 *
*p*	<0.001 *	<0.001 *	

FM, Full Mouth; PD, Pocket Depth; CAL, Clinical Attachment Level; GR, Gingival Recession; PI, Plaque Index; MBI, Marginal Bleeding Index; BOP, Bleeding on Probing; 0, baseline; 1M, examination after 1 month; 3M, examination after 3 months; 6M, examination after 6 months.

**Table 3:** Changes of FM PD, FM CAL, FM GR In groups before (0) and after 1, 3 and 6 months; * *p* < 0.05 [58].

	SRP + i-PRF $\overset{-}{x}$ ± SD	SRP $\overset{-}{x}$ ± SD	*p*
FM PD 0–1 m	0.54 (±0.18)	0.39 (±0.19)	0.011 *
FM PD 0–3 m	0.53 (±0.24)	0.42 (±0.18)	0.25
FM PD 0–6 m	0.49 (±0.31)	0.35 (±0.20)	0.147
FM CAL 0–1 m	0.53 (±0.16)	0.34 (±0.24)	0.003 *
FM CAL 0–3 m	0.50 (±0.26)	0.33 (±0.25)	0.081
FM CAL 0–6 m	0.46 (±0.33)	0.23 (±0.25)	0.038 *
FM GR 0–1 m	−0.006 (±0.02)	−0.04 (±0.12)	0.390
FM GR 0–3 m	−0.03 (±0.08)	−0.09 (±0.17)	0.228
FM GR 0–6 m	−0.03(±0.08)	−0.12 (±0.21)	0.218

FM, Full Mouth; PD, Pocket Depth; CAL, Clinical Attachment Level; GR, Gingival Recession; 0–1, comparison of baseline to examination after 1 month; 0–3, comparison of baseline to examination after 3 months; 0–6, comparison of baseline to examination after 6 months.

**Table 4:** Number of pockets ≥ 5 mm; * *p* < 0.05 [58].

Number of Pockets ≥ 5 mm	SRP + i-PRF $\overset{-}{x}$ ± SD	SRP $\overset{-}{x}$ ± SD	*p*
baseline	23.65 (±16.21)	28.20 (±14.40)	0.175
1M	10.25 (±14.65)	14.20 (±12.68)	0.158
3M	8.05 (±9.47)	13.10 (±11.50)	0.077
6M	9.65 (±11.51)	14.50 (±11.94)	0.095
*p*	<0.001 *	<0.001 *	

1M, examination after 1 month; 3M, examination after 3 months; 6M, examination after 6 months.

**Table 5:** PD, CAL, GR in pockets ≥ 5 mm; * *p* < 0.05 [58].

	SRP + i-PRF SD(±)	SRP SD(±)	*p*
PD_0_	5.56 (±0.45)	5.56 (±0.38)	0.924
PD_1M_	3.91 (±0.90)	4.26 (±0.57)	0.107
PD_3M_	3.89 (±0.45)	4.13 (±0.55)	0.223
PD_6M_	3.96 (±0.57)	4.31 (±0.62)	0.098
*p*	<0.001 *	<0.001 *	
CAL_0_	5.80 (±0.59)	5.77 (±0.54)	0.776
CAL_1M_	4.31 (±0.77)	4.53 (±0.73)	0.49
CAL_3M_	4.17 (±0.66)	4.45 (±0.65)	0.213
CAL_6M_	4.25 (±0.76)	4.68 (±0.72)	0.061
*p*	<0.001 *	<0.001 *	
GR_0_	0.23 (±0.31)	0.21 (±0.35)	0.667
GR_1M_	0.23 (±0.29)	0.26 (±0.41)	0.909
GR_3M_	0.28 (±0.39)	0.32 (±0.47)	0.943
GR_6M_	0.27 (±0.39)	0.38 (±0.55)	0.691
*p*	0.292	0.007 *	

PD, Pocket Depth; CAL, Clinical Attachment Level, GR, Gingival Recession; 0, baseline; 1M, examination after 1 month; 3M, examination after 3 months; 6M, examination after 6 months.

**Table 6:** Difference in PD, CAL and GR of pockets ≥5 mm in groups after 1, 3 and 6 months compared to baseline (0); * *p* < 0.05 [58].

	SRP + i-PRF $\overset{-}{x}$ ± SD	SRP $\overset{-}{x}$ ± S	*p*
PD 0–1M	1.64 (±0.68)	1.29 (±0.42)	0.053
PD 0–3M	1.66 (±0.36)	1.43 (±0.35)	0.069
PD 0–6M	1.59 (±0.55)	1.25 (±0.41)	0.042 *
CAL 0–1M	1.48 (±0.39)	1.24 (±0.52)	0.14
CAL 0–3M	1.62 (±0.38)	1.32 (±0.35)	0.027 *
CAL 0–6M	1.54 (±0.58)	1.09 (±0.52)	0.014 *
GR 0–1M	0.007 (±0.09)	−0.04 (±0.20)	0.938
GR 0–3M	−0.04 (±0.13)	−0.11 (±0.29)	0.546
GR 0–6M	−0.03 (±0.14)	−0.16 (±0.39)	0.342

PD, Pocket Depth; CAL, Clinical Attachment Level; GR, Gingival Recession. 0–1, comparison of baseline to examination after 1 month; 0–3, comparison of baseline to examination after 3 months; 0–6, comparison of baseline to examination after 6 months.

**Table 7:** Mean changes in the MMP-8 levels at the three-month follow-up expressed as ng/mL per 30 s sample; * *p* < 0.05 [58].

	SRP + i-PRF $\overset{-}{x}$ ± SD	SRP $\overset{-}{x}$ ± SD	*p*
MMP-8_0_	23.53 (±20.95)	27.74 (±21.21)	0.456
MMP-8_2W_	4.05 (±5.00)	10.85 (±16.01)	0.208
MMP-8_1M_	15.99 (±24.63)	12.03 (±16.89)	0.999
MMP-8_3M_	9.45 (±17.07)	9.118 (±9.72)	0.125
*p*	0.007 *	0.009 *	

MMP-8, Matrix metalloproteinase-8; 0, baseline; 2W, examination after 2 weeks; 1M, Examination after 1 month, 3M, examination after 3 months.

**Table 8:** Mean changes in the SFFR volume expressed in the relative PU at the six-month follow-up per 30 s sample; * *p* < 0.05 [58].

	SRP + i-PRF $\overset{-}{x}$ ± SD	SRP $\overset{-}{x}$ ± SD	*p*
SFFR_0_	150.45 (±39.72)	140.55 (±52.01)	0.694
SFFR_2W_	79.70 (±48.25)	108.25 (±59.01)	0.96
SFFR_1M_	78.50 (±35.02)	87.53 (±44.45)	0.642
SFFR_3M_	79.40 (±40.91)	90.60 (±42.99)	0.409
SFFR_6M_	76.25 (±38.49)	96.40 (±55.86)	0.279
*p*	<0.001 *	0.015 *	

SFFR, Sulcus Fluid Flow Rate; 0, baseline; 2W, examination after 2 weeks; 1M, examination after 1 month, 3M, examination after 3 months; 6M, examination after 6 months.

## Data Availability

The original contributions presented in this study are included in the article. Further inquiries can be directed to the corresponding author.
